# Supplementary material for: Content-rich biological network constructed by mining PubMed abstracts
Source: BMC Bioinformatics. 2004 Oct 8;5:147. doi: 10.1186/1471-2105-5-147 (PMC528731; doi:10.1186/1471-2105-5-147)
Supplement: Additional File 2 — The original results of the above study (non-essential files are deleted to keep the file size under the limit set by BMC bioinformatics). [file 1471-2105-5-147-S2.bz2 › chilibotAdditionalFile2/dip10/442ID9687537E10418/html/CCR1_CCL14.html]

 


 **CCR1** and **CCL14** 
  
Found 12 abstracts in PubMed, retrieved 10.  
 

 What does Google say? 
 PDF only 
| .edu only 

---

**Interactive relationship** (e.g. stimulation, inhibition, etc)

**Inhibitory relationship**- HCC 1  [ **CCL14** ]  competed with MIP 1alpha for binding to  **CCR1**  transfected cells, but with a markedly reduced affinity IC50 = 93 nM versus 1.3 nM for MIP 1alpha.  Ref: 9687537 J Exp Med, 1998
- Similarly, HCC 1  [ **CCL14** ]  was less potent than MIP 1alpha in inducing inhibition of adenylyl cyclase in  **CCR1**  transfected cells.  Ref: 9687537 J Exp Med, 1998
**Neutral relationship**- These data demonstrate that HCC 1  [ **CCL14** ]  is a chemoattractant and identify  **CCR1**  as a functional HCC 1  [ **CCL14** ]  receptor on human monocytes.  Ref: 9687537 J Exp Med, 1998
- HCC 1  [ **CCL14** ]  9 74 was a potent agonist of  **CCR1** , CCR3, and CCR5 and promoted calcium flux and chemotaxis of T lymphoblasts, monocytes, and eosinophils.  Ref: 11085751 J Exp Med, 2000
- Proteolytic processing of the abundant plasmatic human CC chemokine 1 HCC 1  [ **CCL14** ]  generates a truncated form, HCC 1  [ **CCL14** ]  9 74, which is a potent agonist of  **CCR1** , CCR3, and CCR5.  Ref: 11897579 Antimicrob Agents Chemother, 2002
- Thirty two patients with HCC  [ **CCL14** ] , who had good renal function creatinine clearance Ccr  [ **CCR1** ]  50 ml min or more, received additional intraarterial infusion chemotherapy with LCS or LCS alone.  Ref: 10656904 Cardiovasc Intervent Radiol< MedlineTA>Cardiovasc Intervent Radiol, 2002
- HCC 1  [ **CCL14** ]  induced chemotaxis of freshly isolated human monocytes, THP 1 cells, and  **CCR1**  transfected cells, and the optimal concentration for cell migration 100 nM was approximately 100 fold lower than that of MIP 1alpha 1 nM.  Ref: 9687537 J Exp Med, 1998
- Human CC chemokine 2 HCC  [ **CCL14** ]  2 is a novel member of the chemokine peptide family that induces chemotaxis of monocytes, T lymphocytes and eosinophils via activation of the CCR 1  [ **CCR1** ]  and CCR  [ **CCR1** ]  3 receptors.  Ref: 10604595 J Pept Res, 1999

**Non-interactive relationship** (e.g. studied together, co-existance, homology, etc.)

- We used radioligand binding and 35S GTPgammaS exchange assays in membranes from a cell line transfected to express  **CCR1**  Ba F3 hCCR1 to characterize a panel of chemokines HCC 1  [ **CCL14** ] , MIP 1alpha, MIP 1beta, MIP 1delta, MPIF 1, MCP 2, MCP 3, and RANTES as  **CCR1**  ligands.  Ref: 12381680 Br J Pharmacol, 2002
- Using a cell line expressing CC chemokine receptor CCR  [ **CCR1** ]  5 as a bioassay, we isolated from human hemofiltrate an HCC 1  [ **CCL14** ]  variant lacking the first eight amino acids.  Ref: 11085751 J Exp Med, 2000
- Experiments using a panel of cloned chemokine receptors revealed that HCC 1  [ **CCL14** ]  specifically activated C C chemokine receptor CCR 1  [ **CCR1** ] , but not closely related receptors, including CCR5.  Ref: 9687537 J Exp Med, 1998
- We have previously isolated from human hemofiltrate an N terminally truncated form of the hemofiltrate CC chemokine 1 HCC 1  [ **CCL14** ] , and characterized HCC 1  [ **CCL14** ]  9 74 as a strong agonist of  **CCR1** , CCR5, and to a lower extent CCR3.  Ref: 11544332 J Immunol, 2001
- Identification of C C chemokine receptor 1  [ **CCR1** ]   **CCR1**  as the monocyte hemofiltrate C C chemokine HCC 1  [ **CCL14** ]  receptor.  Ref: 9687537 J Exp Med, 1998
